# Managing acute phantom limb pain with transcutaneous electrical nerve stimulation: a case report

**DOI:** 10.1186/s13256-023-03915-z

**Published:** 2023-05-21

**Authors:** Katleho Limakatso

**Affiliations:** Department of Anaesthesia and Perioperative Medicine, Pain Management Unit, Neuroscience Institute, University of Cape Town, Cape Town, South Africa

**Keywords:** Transcutaneous electrical nerve stimulation, Phantom limb pain, Limb amputation

## Abstract

**Introduction:**

Phantom limb pain is characterized by painful sensations in the amputated limb. The clinical presentation of acute phantom limb pain may differ from that of patients with chronic phantom limb pain. The variation observed implies that acute phantom limb pain may be driven by peripheral mechanisms, indicating that therapies focused on the peripheral nervous system might be successful in reducing pain.

**Case presentation:**

A 36-year-old African male with acute phantom limb pain in the left lower limb, was treated with transcutaneous electrical nerve stimulation.

**Conclusion:**

The assessment results of the presented case and the evidence on acute phantom limb pain mechanisms contribute to the current body of literature, indicating that acute phantom limb pain presents differently to chronic phantom limb pain. These findings emphasize the importance of testing treatments that target the peripheral mechanisms responsible for phantom limb pain in relevant individuals with acquired amputations.

## Introduction

Phantom limb pain (PLP), pain felt in the amputated limb, is a disabling condition associated with depression, decreased mobility, and low quality of life [1]. This condition characterized by painful sharp, shooting, or cramping sensations is estimated to occur in approximately 64% [95% CI: 60.01–68.05] of people who have undergone limb amputations, regardless of the cause of amputation [2, 3]. Phantom limb pain occurs as early as the first day after amputation [4]. However, in some cases the onset may be many months or years after the amputation of a limb [5].

Peripheral and cortical mechanisms for PLP have been proposed [6]. However, it is unclear whether these mechanisms underlie acute or chronic PLP. Acute PLP is classified as pain with an early onset (less than 5 weeks after amputation) and persisting for less than 3 months [4]. Chronic PLP is classified as pain that persists for 3 months or more [4, 7]. Neuroimaging studies of the brains of people with amputations suggest that PLP is driven by neuroplastic changes in the somatosensory, premotor, and primary motor cortices of the brain contralateral to the amputated limb [8–14]. On the contrary, a study by Vaso *et al*. [15] provided compelling evidence that PLP is primarily a bottom-up phenomenon that is initiated by exaggerated input generated ectopically in the dorsal root ganglion of the severed peripheral nerve, and that maladaptive changes in the central nervous system (spinal cord and brain) maybe involved in maintaining the chronicity of pain. The various underlying mechanisms suggest the clinical presentation of people with PLP varies at different stages after amputation.

A substantial difference has been seen in the clinical presentation of acute and chronic PLP [16]. Patients with chronic PLP present (at baseline) with signs and symptoms associated with neuroplastic changes in the sensory and motor areas of the brain: inaccurate left/right judgement scores (< 80%) and/or pain triggered or aggravated by imagined or actual movements of the phantom limb [17]. On the contrary, patients with acute PLP consistently present with accurate left/right judgement scores (> 80%) and report no aggravation of pain with imagined or actual movement of the phantom limb [18–20]. These findings support the existing evidence, suggesting that cortical mechanisms may have a limited role in initiating PLP [15]. Furthermore, this suggests that patients with acute PLP may benefit from treatments targeting maladaptive changes in the peripheral nervous system.

Pharmacological treatments including pregabalin have shown some effect in alleviating PLP [4, 21]. However, the evidence is promising for non-pharmacological interventions such as Transcutaneous Electrical Nerve Stimulation (TENS), which often presents with relatively fewer or no adverse treatment effects [22–25]. Transcutaneous electrical nerve stimulation is a treatment delivered by a battery-powered device via electrodes positioned on the nerve root or along the distribution of the nerve that innervates the painful area [26]. The role of TENS in reducing pain via peripheral mechanisms has been noted in the literature [26]. Given these positive outcomes, TENS might be beneficial for reducing acute PLP in people with amputations. Here we report a case of acute PLP in the left lower limb, treated with a combination of high- and low-frequency TENS.

## Case presentation

A 36-year-old African man, who is 1.8 m in tall, weighs 76 kg, smokes 20 cigarettes per day, and has no prior medical history, was assaulted with a sharp object. He was unconscious upon admission at a tertiary healthcare facility where his left leg was later amputated just below the hip joint. Two days after the amputation, the patient reported excruciating PLP along the length of his missing leg and toes. He reported a pain severity of 7/10 (on a 0–10 scale) and described the pain as shocking and cramping—as if the leg was being twisted. His pain was constant throughout the day and night, and without any notable relief. To manage his pain, he was initiated on Lyrica (25 mg during the day; 150 mg at night), venlafaxine (75 mg), and ibuprofen (200 mg). However, after seven days of treatment, there was no significant improvement in his symptoms. He was referred to the Pain Clinic at Groote Schuur Hospital for reassessment and management of acute PLP.

On assessment, the Douleur Neuropathique four questions (DN4) questionnaire for neuropathic pain revealed a score of 4 out of 10, thus indicating the presence of neuropathic pain [27]. In this questionnaire, he reported symptoms such as hypesthesia to touch, electric shocks, numbness, and itching of the stump.

The overall pain severity score assessed by the pain severity scale of the Brief Pain Inventory (BPI) was 5.5 (on a 0–10 scale) [28]. The individual components of the BPI showed that his pain (out of 10 in the last 24 hours) was five at its worst, four at its least, five on average, and five at the time of assessment. The pain interference score assessed using the pain interference scale of the BPI was five (on a 0–10 scale). Pain had a substantial negative impact on his sleep (9 out of 10) and his walking ability with crutches (7 out of 10), and had minimal interference with general activity (4 out of 10), mood (3 out of 10), relations with other people (2 out of 10), and enjoyment of life (3 out of 10). Because he was an inpatient, we could not rate the interference of pain with normal work. Therefore, the overall pain interference score was derived from six items of the pain interference scale.

The patient reported primary hyperalgesia but no allodynia near the site of amputation. The visual inspection of the stump showed redness and swelling. On left/right judgements he scored: left limb 98%, time 1.4 seconds; right limb 100%, time 1.5 seconds. Imagined and actual movements (knee flexion/extension) of the phantom limb did not aggravate pain. The Tinel's test on the residual limb elicited a shocking pain radiating down the phantom leg into the toes.

**Fig. 1:**
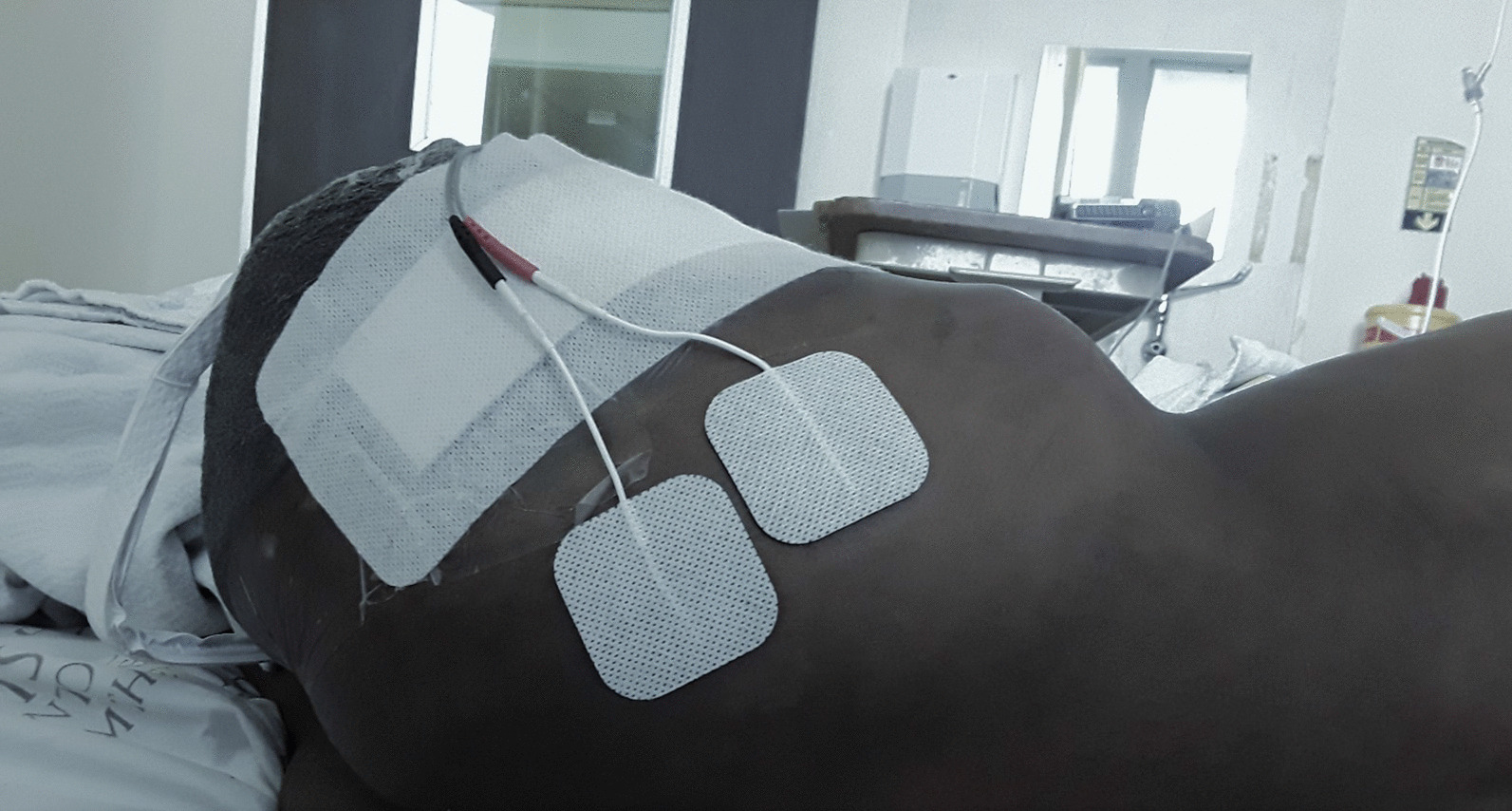
The patient undergoing high- and low-frequency transcutaneous electrical nerve stimulation

Treatment began with educating the patient about PLP and its underlying peripheral mechanisms. He was told in lay terms that spontaneous nociceptive activity at the site of the severed nerve may have a role in initiating PLP and that TENS may provide pain relief. The patient underwent high-frequency TENS (100 Hz) for 15 minutes, followed immediately by 15 minutes of low-frequency TENS (10 Hz). In both instances, the intensity was gradually increased three times to the highest tolerable level. The electrodes were positioned on the posterolateral aspect of the residual limb along the distribution of the sciatic nerve (Fig. 1). At the end of the session, the patient reported complete pain relief and increased awareness of the phantom limb. In addition, the patient reported a high level of satisfaction with the treatment and its effects.

Treatment was provided once a day for three consecutive days, following which outcomes were reassessed. The patient reported no PLP. Further, he reported that his sleep had improved remarkably since the first treatment session. At this point, he was mobilizing with elbow crutches under supervision. No adverse effects were reported.

## Discussion

This is a case report of a patient with PLP in the left lower limb, who was treated with high- and low-frequency TENS. The results of this case indicate that TENS may be effective for reducing acute PLP and its interference with sleep and mobility.

Previous studies on PLP indicate that acute and chronic PLP have been managed using the same approach, which unsurprisingly yielded mixed findings [29–32]. For example, although some people with chronic PLP may benefit from treatments targeting central mechanisms (e.g., mirror therapy), it appears that individuals with acute PLP do not derive any significant benefits from these treatments [32, 33]. This highlights the importance of differentiating between the two PLP classifications and utilizing treatments that address mechanisms underlying each pain type.

Recent neuroimaging evidence has linked PLP to maladaptive changes in the somatosensory, premotor, and motor cortices of the brain—where the neighboring cortical areas shift into the cortical area that previously innervated the amputated limb [34]. The maladaptive changes in these areas are associated with low left/right judgement scores [decreased accuracy (< 80%) and increased recognition time (in seconds)] and aggravated pain with imagined or actual movements of the phantom limb [35–38]. In this case, however, the patient presented with almost perfect left/right judgement scores (left 98%, time 1.4 seconds; right 100%, time 1.5 seconds). In addition, he did not report aggravated pain during the imagined or actual movements of the phantom limb. Therefore, the results of this assessment suggest that cortical mechanisms proposed to drive chronic pain may have a limited role in acute PLP.

The peripheral afferent theory of PLP suggests that increased ectopic firing from an injured nerve is the generator of acute PLP, and potentially a driver of secondary central changes linked to chronic PLP [39]. In fact, the mechanistic study by Vaso *et al*., [15] provided evidence showing that PLP after amputation may be triggered by increased nociceptive activity in the dorsal root ganglion of the severed peripheral nerve. These findings corroborate those of a previous study suggesting that acute PLP is triggered by increased production of substance P and calcitonin gene-related peptide in the dorsal root ganglion and maintained by exaggerated nociceptive activity between the first and second order neurons in the dorsal horn of the spinal cord [40, 41]. Further, it is known that triggering nociceptive activity at the nerve site evokes symptoms distally in regions that are innervated by that particular nerve. A positive Tinel's test in this case highlights the peripheral nerve as an important treatment target.

High-frequency (100 Hz) TENS has been shown to provide analgesia by activating the gate-control mechanism via the fast-conducting, heavily myelinated A-beta fibers that compete with the transmission of nociception from the periphery by the slow-conducting C fibers [42]. Low-frequency TENS (10 Hz) provides analgesia by activating the opioid receptors in the periphery and dorsal horn of the spinal cord [26]. Although there is some preliminary evidence suggesting the mechanisms by which TENS reduces acute PLP, further mechanistic studies are necessary to elucidate this association.

Transcutaneous electrical nerve stimulation has consistently shown positive results in patients with neuropathic pain syndromes [23, 43]. In addition, the treatment is inexpensive, and it requires minimal patient training [44]. The effectiveness of TENS coupled with its safety and a lack of adverse effects makes it a suitable complementary analgesic intervention for patients with acute PLP.

## Conclusion

The results of the assessment of the presented case and the neurophysiological evidence on acute PLP mechanisms adds to the existing literature indicating that acute PLP presents differently to chronic PLP. This, therefore, highlights the need for testing the efficacy of treatments targeting peripheral mechanisms underlying acute PLP in people with acquired amputations.

## Data Availability

The participant’s de-identified data will be made available upon reasonable request.

## Abbreviations

PLP: Phantom limb pain
TENS: Transcutaneous electrical nerve stimulation
